# The beauty and the beast of social media: an interpretative phenomenological analysis of the impact of adolescents' social media experiences on their mental health during the Covid-19 pandemic

**DOI:** 10.1007/s12144-023-04271-3

**Published:** 2023-01-21

**Authors:** Betul Keles, Annmarie Grealish, Mary Leamy

**Affiliations:** 1 Florence Nightingale Faculty of Nursing, Midwifery and Palliative Care, King’s College London, James Clerk Maxwell Building, Room 1.38a, 57 Waterloo Road, London, SE1 8WA UK; 2 Department of Nursing and Midwifery, Faculty of Education & Health Sciences, University of Limerick, Room HS3-032, Health Sciences Building, Limerick, V94 T9PX Republic of Ireland

**Keywords:** Adolescents, Social media, Covid-19, Mental health, Qualitative study, Risk and protective factors

## Abstract

Despite extensive research, the mental health implication of social media in adolescents is not yet understood due to mixed and inconsistent findings and more in-depth qualitative studies are needed to expand our understanding of the impact of social media on adolescent mental health during the Covid-19 pandemic. The purpose of this study was to explore why and how adolescents use social media, adolescents’ lived experiences on social media, how they make sense of these experiences having impact on their mental health, and the influence of the Covid-19 pandemic on their use of social media and mental health. In-depth interviews were conducted with eleven adolescents aged 14–16 (five female, six male) across England. The interviews were audio-recorded, transcribed verbatim and analysed using interpretative phenomenological analysis. Two key themes were identified: the beauty of social media that captured positive experiences and emotions of adolescents and the beast of social media that captured negative experiences and emotions. From the adolescents’ accounts, social media has both positive and negative impacts on their mental health, but mostly positive impacts during the Covid-19 pandemic. The results were discussed in relation to the study aims and previous study findings. Strengths and methodological limitations of the study, implications for future research that emerged from the study were discussed.

## Introduction

In the last ten years, the number of social media users has rapidly increased from 0.97 billion in 2010 to 3.40 billion in 2019 (eMarketer, 2017; Statista, 2021). Since the start of the Covid-19 pandemic in March 2020 and restrictive measures being introduced 2020–2021 to contain the spread of the virus, the number of social media users worldwide has soared further by 23% which reached 4.20 billion active social media users (Johnson, 2021). According to national UK and international data, the most enthusiastic users of social media were adolescents (Abraham, 2020; Lenhart, 2015).

Research on social media use and its impact on the mental health of adolescents has increased recently, with many studies investigating whether constant use of social media is associated with mental health problems, including anxiety and depression (e.g., Karim et al., 2020; Marino et al., 2018; Nesi, 2020; Twenge & Martin, 2020). Despite extensive research, the effect of social media on the mental health of adolescents is not yet understood due to mixed and inconsistent findings (Best et al., 2014; Keles et al., 2020; Orben, 2020; Schønning et al., 2020; Valkenburg et al., 2021). Considering the mixed findings (i.e., no effect, positive, negative or both positive and negative effects) in the literature which mainly came from cross-sectional quantitative studies, there is a clear need for in-depth qualitative studies to expand our understanding of why and how adolescents use social media and how social media impacts upon their mental health (Keles et al., 2020; Schønning et al., 2020).

To date, a few studies explored adolescents’ perspectives and experiences of social media using a qualitative approach. For example, O’Reilly et al. (2018) conducted six focus group interviews involving 54 UK adolescents aged 11–18 and revealed only negative influences of social media (i.e., mood and anxiety disorders, cyberbullying, and addiction) from the perspectives of adolescents. In contrast, other studies (e.g., Radovic et al., 2017; Weinstein, 2018; O’Reilly, 2020; Fiacco, 2020; Hjetland et al., 2021) highlighted that the mental health impact of social media is two-sided; from the perspectives of adolescents, social media has both positive and negative influences on their mental health. The summary findings of these studies are illustrated in Table 1.

**Table 1:** Summary of findings from qualitative studies exploring adolescents’ perspectives and experiences of social media

Authors (year)	Setting	Participants	Method	Positive findings	Negative findings
Radovic et al. (2017)	US	23 adolescents aged 13–20	In-depth interviews	Searching for positive content Connection	Sharing risky behaviours Cyberbullying Social comparison
O’Reilly et al. (2018)	UK	54 adolescents aged 11–18	Focus group interviews	–	Mood and anxiety disorders Cyberbullying Addiction
Weinstein (2018)	US	26 high school students from grade 9, 10 and 11	In-depth interviews	Interactions with others (closeness vs disconnection) Self-expression (affirmation vs concerns about others’ judgements) Interest-driven exploration (inspiration vs distress) Online browsing (happiness vs boredom; admiration vs envy)	
O’Reilly (2020)	UK	54 adolescents aged 11–18 and mental health practitioners	Focus group interviews	Connectivity/ Communication Resilience to stress	Addiction Fear of missing out Social comparison Poor sleep Cyberbullying Exposure to content that promotes self-harm
Fiacco (2020)	US	9 adolescents aged 13–15	In-depth interviews	Connection communication Sense of belonging Positive feedback Entertainment	Upward and downward social comparison Cyberbullying Distressing content
Hjetland et al. (2021)	Norway	27 adolescents aged 15–18	Focus group interviews	Staying connected	Addiction Social comparison Cyberbullying Fear or missing out Fear of being left out Permanency of online content

Collectively, beneficial effects of social media are linked to its use as a means of connecting with others, especially though enabling easier connection and communication with families, friends, and new people (Anderson & Jiang, 2018a, b; Clark et al., 2018). It is well-established that social connectedness and a sense of belonging are linked to a low risk of anxiety and depression among adolescents (Allen et al., 2014; Jose et al., 2012; McLoughlin et al., 2019). Therefore, online connection and communication can facilitate social and emotional support (Michikyan & Suárez-Orozco, 2016; Weinstein, 2017), which can then enhance the mental health of adolescents and reduce the risk of mental health problems including anxiety, depression and self-harm (Nesi, 2020).

Adolescents and young people have an intrinsic desire to obtain other’s approval through expressing themselves in an idealized way (McLeod & Genereux, 2008; Rudolph et al., 2005), and social media provides opportunity to receive feedback from others through self-expression (Boyd, 2007; Stern, 2008). An analysis of data from 10,560 Facebook users revealed that authentic self-expression on social media was associated with greater subjective well-being (Bailey et al., 2020). Social media also enables young users to learn and access to information they need (O’Keeffe et al., 2011) and receive social support which can protect adolescents against distress and facilitate resilience to stress (Ozbay et al., 2007; Zautra et al., 2010). In addition, social media may serve as a tool to satisfy adolescents' needs for gratifications during the Covid-19 pandemic and may protect adolescents against psychological distress caused by the pandemic. Nevertheless, previous literature tends to claim that the use of media as a tool for coping with stress is problematic because escaping and denying the daily life stresses temporarily creates a boundary between the individual and the reality, but in the long term it can increase the experience of distress (e.g., Carver & Connor-Smith, 2010; Müller et al., 2016).

Conversely, social media use is also associated with harmful and unintended consequences, for example excessive use of social media increases the risk of being addicted to it. Many studies have found a statistically significant relationship between social media use addiction and the symptoms of depression and anxiety (Keles et al., 2020; Kelly et al., 2018). Studies have also shown that exposure to negative media that promote violence can cause antisocial and aggressive behaviours among adolescents (Anderson et al., 2003; Bushman & Anderson, 2009; Lin, 2013; Savage, 2004). Moreover, social media is one of the most common places that cyberbullying can occur. Cyberbullying can be more prevalent than traditional bullying as it can take place anywhere and anytime in a range of forms, including insults, threats, spreading rumours, sending explicit texts or images, and exclusion (excluding someone from activities) (Peebles, 2014). Studies have shown that cyberbullying is strongly correlated with the risk of self-harm and suicidal behaviours (e.g., John et al., 2018; Kuehn et al., 2019) as well as internalizing (e.g., anxiety, depression, anger) and externalizing problems (e.g., risk taking behaviours, alcohol and substance use) (Fisher et al., 2016) in adolescents. Other possible harms associated with social media use include its impact on sleep patterns. It is well-known that adequate and quality sleep is important for adolescent development and mental health (Orchard et al., 2020; Tarokh et al., 2016; Vredenburgh, 2017). However, some evidence has shown that adolescents were likely to constantly check social media at night, and have an urge to respond to messages quickly, which can lead to poor sleep which in itself may increase the risk of anxiety and depression (Kelly et al., 2018; Scott et al., 2019; Woods & Scott, 2016).

Social media can also trigger the fear of missing out which is an unpleasant feeling characterized by anxiety about missing online content and interactions from others and the inability to respond in a timely fashion (Alutaybi et al., 2020; Barry et al., 2017). For example, adolescents may experience fear of missing out when they see posts of their friends on social media going out socialising where they were not invited to (Abel et al., 2016). Finally, according to the social comparison theory, people have a fundamental desire to compare themselves with others to evaluate their opinions, abilities, attitudes, physical appearances and their life (Dijkstra et al., 2008; Festinger, 1954). There is no doubt that social media enables users to make social comparisons with others. Making social comparisons on social media can cause body image concerns, eating problems, and having an unrealistic view of others’ lives, which may then lead to mental health problems (e.g., Fardouly et al., 2020). It is thought that this is because social media platforms allow users to post the best moments of their lives and their most attractive photos after they have been filtered and edited, leading to adolescents making detrimental comparisons of their own lives and physical appearances to these edited and enhanced photos. As a result of such negative social comparisons, they can feel worse about their own lives and their physical appearances, which in turn may lead to poor body image and mental health issues (Holland & Tiggemann, 2016). Evidence has shown that negative social comparison was positively correlated with depression in adolescents (Fardouly & Vartanian, 2016).

### The current study

Social media may influence adolescent mental health in both positive and negative ways. However, the qualitative studies cited above were published before the Covid-19 pandemic, and thus they did not explore the impact of social media on adolescent mental health during the pandemic. The global pandemic has caused individuals to face social and economic losses and the uncertainties of new living conditions. This has led individuals to face a new reality where the perceived role of social media may have changed because social media has become a primary tool that fulfils individuals' needs for connection with the world, communication with family and friends, socialization, entertainment, and information. From the previous studies, it remains unclear whether and to what extent social media leads to positive or negative mental health consequences for adolescents during the Covid-19 pandemic. Covid-19 pandemic may have heightened the positive and exacerbated the negative impacts of social media or vice versa.

This study, therefore, addresses some of the gaps in evidence by reporting on a study that aimed to explore why and how adolescents use social media, adolescents’ lived experiences on social media, how they make sense of these experiences having impact on their mental health, and the influence of the Covid-19 pandemic on their use of social media and mental health.

## Method

### Study design

To address this study's aim, a qualitative study design using Interpretative Phenomenological Analysis (IPA) (Smith & Osborn, 2003; Smith et al., 2009) was used to capture adolescents’ lived experiences on social media and understand their views and perceptions about the influence of these experiences on their mental health. This research is reported in accordance with the consolidated criteria for reporting qualitative research (COREQ) checklist (Tong et al., 2007).

### Ethical considerations

Ethical approval for the study was granted from the Psychiatry, Nursing and Midwifery Research Ethics Subcommittee at King’s College London (Ethics Ref: HR-19/20-20856). All participants were clearly informed about the purpose of the study, their rights explained in relation to confidentiality, the voluntary nature of participation, with all potential participants being informed that they were free to participate, refuse or withdraw at any time. Parental consent was also obtained from the parents/guardians of the participants under 16 years. Potentially identifying details of all participants were removed during the transcription, anonymity was assured by using pseudo name instead of participants names.

### Participant selection and recruitment

**Fig. 1:**
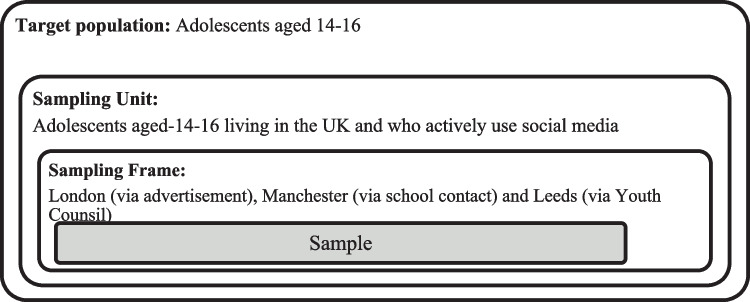
Sampling frame

In keeping with IPA sampling requirements, a small purposive homogeneous sample of adolescents aged 14–16 were recruited using the following eligible criteria: (i) aged 14–16, (ii) actively engaging with social media, and (iii) living in the UK and (iv) able to speak English. Sampling frame was illustrated in Fig. 1.

A sample size of 8–10 potential participants was aimed for because IPA studies focuses on the particular individual in a particular context and a detailed account of their experience is said to be sufficient, typically up to 10 participants is sufficient (Smith et al., 2009). In this study, eleven participants were recruited over 7-months (October 2020 to May 2021) through advertisement on the XXX research recruitment page (*n* = 9), a secondary school in the UK (*n* = 1), and Leeds Youth Council (*n* = 1). The lead author screened participants who expressed an interest in participating against the eligibility criteria, and if they were eligible asked them, when necessary, their parents or guardians, to provide written informed consent/ascent via email. There was no established relationship between the interviewer/lead author, and any of the participants before the interviews were conducted.

### Data collection

Data were collected using semi-structured interviews guided by an interview guide. Due to the Covid-19 restrictive measures on face-to-face contact, video meetings were arranged with each participant individually using Microsoft Teams and only the interviewer and the participant were present. The interview guide was developed collaboratively with three experienced clinicians in Child and Adolescent Mental Health Services (CAMHS). It was then piloted with a 14-year-old male adolescent and an undergraduate children's nursing student, resulting in only minor adjustments to the flow and order of the questions, were made. The interview guide was designed to be used flexibly, with a combination of open-ended questions and follow-up probes to facilitate exploration of participants’ own personal experiences and included questions about a) why adolescents use social media, b) what they do on social media, c) how they make sense of the impact of social media on their mental health, d) how the Covid-19 pandemic impacted on their social media use and mental health, and e) how they make sense of the impact of social media during the pandemic. All participants received a £10 Amazon e-gift card as a thank you for their participation.

All interviews were audio recorded and the lead author kept a reflective diary and notes from the interviews, aiding the analytic process. The mean interview duration was 60 min (ranging between 27–103 min). A total of 672 min of interview material was collected. All interviews were transcribed verbatim by the lead author to help immersion in the data, anonymised to ensure confidentiality and stored in a password-protected encrypted database.

### Data analysis

Transcribed interviews were uploaded to NVivo 12 for data management purposes and to perform the qualitative coding procedure. Transcripts were analysed following IPA six-step process outlined by Smith et al. (2009) which consisted of: (1) transcripts were read and re-read several times to ensure the essence of each narrative was captured; (2) initial notes were taken; (3) emergent themes were identified and coded; (4) connections across emergent themes were identified to cluster them into super-ordinate themes; (5) the same process was repeated for each transcript; (6) similarities and differences across all of the narratives were identified to develop final set of themes. While the initial analysis was performed by the lead author, to enhance rigor, the lead author’s two supervisors who have extensive experience in conducting qualitative research, critically examined the transcripts with preliminary coding, original notes, theme tables, the process of theme development and independently coded 25% of the transcripts. The lead author and her two supervisors engaged in extensive debate on, and critique of, each other’s dataset interpretations and reviewed each stage, with any disagreements discussed until consensus was reached. Interviews were stopped when the research team considered the dataset’s ability to sufficiently illuminate participants’ explicit meaning-making and when dataset richness was deemed sufficient. As per guidance recommended by the IPA originators (Smith & Osborn, 2003) appraisal of the richness of the dataset determined when data collection stopped.

### Rigour

The methodological rigour of this qualitative research was established through the implementation of “the Four-Dimensions Criteria” (credibility, dependability, confirmability, and transferability) proposed by Lincoln and Guba (1985). To ensure the analysis was sufficiently rigorous and minimised the potential for bias, the lead author conducted a pilot test of the interview guide and kept a reflexive diary during data collection and analysis stages. The dependability was reinforced by strategies such as publishing a detailed study protocol and ensuring a high level of consistency, and inter-coder agreement within the research team. To aid the process of bracketing-off and ensure interpretations were grounded in the participant’s words, the lead author received regular supervision with the research team, including discussions around research participants and settings, obtaining adequate participation, transcribing the interviews verbatim, and using a recognized data analysis method. This also contributed to confirmability and transferability in this study.

## Findings

### Sample demographics and social media use characteristics

Eleven adolescents were interviewed from different regions across England (London (*n* = 9), Manchester (*n* = 1), Leeds (*n* = 1). Participant characteristics are presented in Table 2. Participants were aged 14 to 16 years, mean age of the 11 adolescents was 15. All of the adolescents lived in the same household with one or both parents. The ethnicity of the adolescents (*n* = 11) varied, just over a third were British/Irish (*n* = 4), Finnish (*n* = 2), Pakistani (*n* = 2), Brazilian (*n* = 1), Portuguese (*n* = 1), and Vietnamese (*n* = 1). The most used social media platforms among the adolescents were Snapchat (*n* = 10), Instagram (*n* = 9), TikTok (*n* = 5), WhatsApp (*n* = 3), YouTube (*n* = 1) and Twitter (*n* = 1). The motives for using social media included communication, entertainment, information seeking, ease of use, content sharing, need to belong, and connection. Although all eleven adolescents engaged with both active and passive social media use behaviours, ten adolescents reported they often remain passive on social media and prefer to scroll through their feed and look at other users’ activities rather than posting anything.

**Table 2:** Sample demographics and social media use characteristics

Made-up name	Age/ Gender	Nationality	Family background	Length of social media membership	Social media platforms they use	Motives for social media use	Privacy setting	Daily time spent	Number and the type of followers	Type of accounts they follow
Orla	15 years; female	British-Pakistani	Has 3 siblings Mother: University graduate/employed Father: High-school graduate/self-employed	5 years	Instagram Snapchat TikTok	Easy to use Entertainment Information Communication	Private	3 h	300/ Family, friends Strangers	Family, Friends, Celebrities, Entertainment/ Meme accounts
Sofia	16 years; female	British	Has 2 siblings Mother: University graduate/unemployed; Father: High-school graduate/employed	6 years	Instagram Snapchat TikTok	Communication Need to belong	Public	11 hr	295/ Family, friends	Family, Friends, Celebrities Community pages
Alice	16 years; female	Brazilian	Has 1 sibling Mother: University graduate/employed Father: High-school graduate/employed	3 1/2 years	Instagram TikTok	Entertainment Information Connection Communication Content sharing	Private	3 h	721/ Family, friends	Family, Friends, Celebrities
Oliver	14 years; male	British-Portuguese	Has no sibling Mother: University graduate/student Father: High-school graduate/unknown	2 1/2 years	Snapchat YouTube WhatsApp	Entertainment Communication	Private	< 1 h	17/ Family, friends	Family, Friends, Sports accounts
Clara	15 years; female	British	Has 1 sibling Mother: University graduate/employed Father: University graduate/employed	2 years	Snapchat TikTok	Communication Content sharing	Private	2 h	600/ Family, friends	Family, Friends, Celebrities, Dancers, Brands
Ralph	14 years; male	Finnish	Has 1 sibling Mother: University graduate/employed Father: University graduate/employed	2 years	Instagram Snapchat TikTok	Easy to use Entertainment Information Need to belong	Private	3 h	500/ Family, friends	Family, Friends, Influencers, Entertainment/ Meme accounts
Arthur	16 years; male	British-Vietnamese	Has 1 sibling Mother: High-school graduate/self-employed Father: University graduate/self-employed	2 years	Instagram Snapchat	Easy to use Need to belong	Private	2 h	288/ Family, friends Strangers	Family, Friends, Celebrities, Community pages
Scarlett	16 years; female	Finnish	Has 1 sibling Mother: University graduate/employed Father: University graduate/employed	4 1/2 years	Instagram Snapchat	Entertainment Communication	Public	3 h	1300/ Family, friends Strangers	Family, Friends, Sports accounts, Entertainment/ Meme accounts
Jack	15 years; male	British	Has 2 siblings Mother: University graduate/employed Father: University graduate/unemployed	1 1/2 years	Instagram Snapchat Twitter	Entertainment Communication Information	Private	6 h	600/ Family, friends	Family, Friends, Celebrities, Sports accounts, Entertainment/ Meme accounts, News account
Charlie	16 years; male	British	Has 2 siblings Mother: University graduate/employed Father: University graduate/employed	1 1/2 years	Instagram Snapchat WhatsApp	Connection Communication	Private	1 h	200/ Family, friends	Family, Friends, Celebrities
Daniel	15 years; male	British-Pakistani	Has 2 siblings Mother: University graduate/unemployed Father: High-school graduate/employed	2 years	Instagram Snapchat WhatsApp	Need to belong Communication Information	Private	1 h	80/ Family, friends	Family, Friends, Celebrities, Sports accounts, Entertainment/ Meme accounts
Number of participants										11
Age Range Mean Age										14 to 16 years 15.27
Male: Female ratio										6:5

The average length of social media membership was 3 years, ranging from one and a half years to six years. Adolescents’ average time spent on social media during the day was 3 h, ranging from 30 min to 11 h per day. From the accounts of adolescents, Covid-19 quarantine measures (e.g., staying at home, social distance, reduced social interactions/activities), excessive free time, endless scrolling feature of social media and addictive content increased the time they spent on social media, while parental control, being busy with other work (e.g., schoolwork), outdoor activities (e.g., meeting with friends) reduced the time they spent on social media. Nine adolescents reported that they used social media in privacy setting, so that allowed only their families and friends were able to access their profiles. The reasons they gave for using a private setting included not needing to show others what they are doing, preventing random people from following them, seeing their posts and sending message requests. Two adolescents reported they had set their social media profiles to a public setting because they do not post anything on social media.

### Main findings

**Fig. 2:**
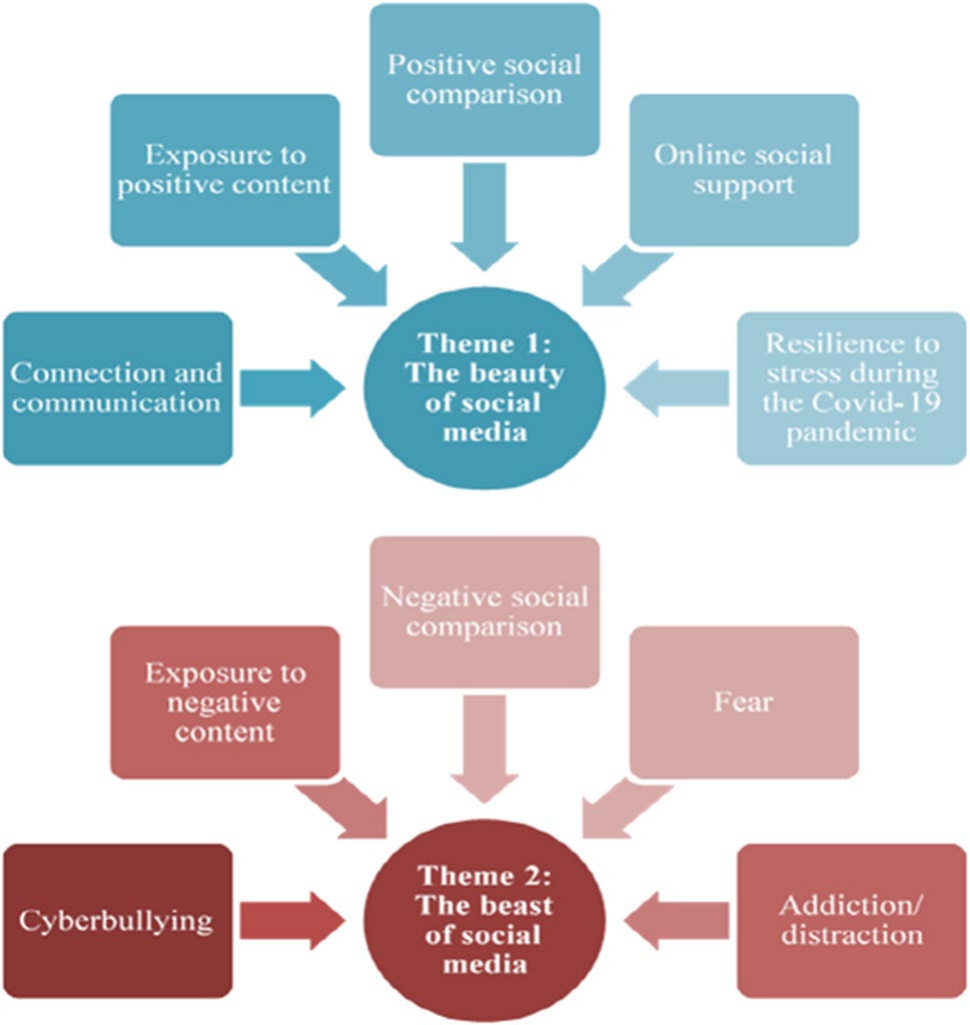
A schematic overview of the main themes (circles) and subthemes (squares)

Two superordinate themes emerged from participant data: 1) The beauty of social media and 2) the beast of social media. Each of these themes was originated from several subthemes shown in Fig. 2. The subthemes were presented using direct quotes from the adolescents to ensure transparency, consistent with the IPA research methodology. All quotes were labelled with adolescents’ pseudonyms and age.

### Theme 1: beauty of social media

The first superordinate theme ‘Beauty of social media’ that emerged in all adolescents’ interview conveys the positive experiences and emotions that adolescents experienced during their use of social media. This theme included five sub-themes: connection and communication, exposure to positive content, positive social comparison, online social support, and resilience to stress during the Covid-19 pandemic.

#### Connection and communication

Social media was described as a medium that facilitates connection and communication between people, which was one of the main reasons for using social media among most adolescent participants and considered as one positive aspect of social media. The importance of connection and communication with family, friends, and new people, especially during the Covid-19 pandemic when face-to-face communication was not allowed and its positive influence on their mood was discussed in some form by almost all adolescents. From adolescents’ perspectives, being able to contact friends on social media made them feel happy and stopped them from being worried and overthinking. This indicates that connection and communication were important factors for adolescent mental health.

*“It helps my mood a lot of the time because I obviously talk to my friends about anything that might be stressing me out or making me uncomfortable.” (Scarlett, 16)*

#### Exposure to positive content

Exposure to positive content (i.e., information, image and/or video) that enables adolescents to discover, learn, have fun and become aware of the things happening in the world was considered as another positive experience of adolescents on social media. Participants expressed that they felt better, happy, and excited when they saw their friends posting, positive things happening in the world, funny contents, and new things they discovered on social media such as information, music, art, and fashion.

*“I see good art and I get happy.” (Arthur, 16)*

From the account of Orla, entertainment was one of her main motives for using social media, and thus exposure to funny content on social media did not only make her happy but also elevated her mood when she felt sad:

*“If you are sad and you go into like TikTok or Instagram and you just see like loads of funny stuff happening then it can make you like quite happy.” (Orla, 15)*

Also, as the 'Black Lives Matter' movement was a relatively new and quite influential movement at the time of the interviews, referring to this incident, adolescents perceived social media as a ‘powerful’ tool that gather millions of people under an umbrella for one purpose. Adolescents expressed that social media was helpful in raising awareness by showing what is happening around the world and creating a sense of unity against the wrong things happening in the world.

*“A lot of people were sharing out on their stories. That was helpful because it kind of you get to find out a lot of information from the post that people put up.” (Jack, 15)*

#### Positive social comparison

Positive social comparison can lead to several positive consequences including self-esteem, self-image, well-being, and self-motivation. Adolescents expressed how engaging with horizontal social comparison on social media helped them to develop a positive self-image and made them feel better and more confident when they see others looking similar to them (i.e., same ethnicity) or having similar flaws in their appearances (i.e., big nose).

*“When I am on TikTok and I saw like a lot of people had a similar nose to me and that they were still happy with their nose. If you get what I mean like it made me feel a bit better about my nose” (Orla, 15)*

Some adolescents also reported making upward social comparisons (i.e., comparing themselves with someone who has achieved what they desire to achieve) in a positive manner for the purposes of ‘self-motivation’ made them feel motivated toward their goals and improve themselves.

*“It is cool that they have, um, they’re, you know, in really good shape, but it’s more like I want to go towards that direction, but it does not really make me feel negatively about myself.” (Scarlett, 16)*

#### Online social support

For adolescents, social media was also perceived as another way of getting social support which is often linked to an improved mental health. From the accounts of adolescents, being mentioned on a page with a large number of followers, getting compliments from others on a shared content, and getting loads of advice were considered as positive experiences associated with social media, and gave them a positive feeling.

*“If you post something online and people are just sent like saying like those are compliments and something like your praise and that then that will affect you positively” (Orla, 15)*

#### Resilience to stress during the Covid-19 pandemic

Adolescents reported that the Covid-19 pandemic impacted their mood in a negative way. Most of these adolescents described their experiences within national lockdowns and they expressed that lockdown measures and their consequences (i.e., staying home, not being able to go out and meet with friends, missing subject matter units, and being unable to perform daily activities as usual) made them feel worried, distressed, and bored.

*“The coronavirus with lockdown affected my mood, impacted my mood with like nervousness like making me worried, making me actually bored, stuck at home and stuff.” (Oliver, 14)*

However, social media helped adolescents cope with the negative impacts of the Covid-19 pandemic and restrictions on their mood by satisfying their needs for connection and communication, entertainment, getting news and learning information. Especially during the national lockdowns, when restrictions were in place and face-to-face communication was not allowed, social media has given adolescents the opportunity to connect and communicate virtually with their friends and other family members. This was especially important for adolescents because they were not able to go out, socialize and entertain themselves during the national lockdowns. The following account from Charlie captures this:

*“Because we are isolated, we can't physically hang out with our friends or families. So, social media has acted as like a medium for staying in contact with people and our friends and family.” (Charlie, 16)*

Also, thanks to social media, they felt better and more adaptive to the situation they were in when they saw other people who were experiencing the same situations as they were (e.g., staying at home during national lockdown).

*“I think it [social media] did help because you know everyone was posting their experiences and then everyone was saying, you know, how bored the national lockdown was. It was not only me going through this. Everyone is going through it.” (Daniel, 15)*

Some adolescents also described their distress, sadness, and fear when they were exposed to news about the number of Covid-19 cases and deaths and the fight against the pandemic worldwide. On the other side, for some adolescents, social media was the first place that they have heard about the positive news about the Covid-19 pandemic. This indicates that social media meets adolescents’ needs for information, which gives adolescents a sense of ‘positivity’. The following account captures this and associated feelings:

*“I found about the vaccine through social media. I didn't need to check the news then and everyone’s posting about it. That gave me some positivity.” (Daniel, 15)*

### Theme 2: the beast of social media

The second superordinate theme ‘The beast of social media’ that emerged for nine adolescents reveals adolescents’ negative experiences on social media and their perspectives on how these experiences have a detrimental impact upon their mental health. Five subthemes grouped under this theme included negative content, cyberbullying, negative social comparison, fear, and addiction/distraction.

#### Exposure to negative content

Negative contents, which adolescents talked about, included written and/or visual contents that include inappropriate/rude content, violence against people and animal, distressing political issues and that promote discrimination and hate towards one’s gender, race, and political views. Adolescents described their experiences of ‘sadness’, ‘distress’ and ‘frustration’ towards such contents. The following account from Clara captures her feelings towards distressing contents she exposed on social media:

*“It has made me feel sad because I look through things that are like really sad news [on social media] about Covid or a bombing or a political thing going on or war, or people who can't afford thing. And that does upset me a lot. (Clara, 15)*

#### Cyberbullying

Adolescents felt sad, angry, and annoyed when they were in a conflict with someone on social media and when they were exposed to cyberbullying behaviours such as receiving distressing and malicious comments/messages online. This form of bullying was particularly annoying because they were not able to fight back with their bullies behind the screen.

*“I felt annoyed 'cause I cannot do anything to them 'cause they're behind the screen and I was behind the screen as well.” (Ralph, 14)*

*“I felt pretty horrible about it, you know. I cried myself for a bit.” (Arthur, 16)*

From the quotations, it is evident that the experience of cyberbullying had a negative impact on adolescents' mental health. Having difficulty in coping with cyberbullying behaviours and its impact on their self-image was coupled with a sense of extreme sadness and distress.

#### Negative social comparison

Adolescents described their experiences of social comparison and how these experiences had a negative impact on their mood. From the adolescents’ responses, it was observed that their negative experiences were mostly caused by upward social comparison rather than downward or horizontal ones. The social comparison areas were diverse, and these areas included physical appearance, lifestyle, fashion, and style. The most prominent emotion that emerged was envy when adolescents compared themselves with people who they think have a better and luxurious life than they have:

*“It makes you a bit jealous to see their massive houses in there or their nice cars and stuff” (Jack, 15)*

Adolescents, especially female adolescents, appear to compare themselves with others in terms of weights, body shape and appearance. From the female adolescents’ accounts, social media triggered their body image concerns as they were exposed to maximally attractive photos of other female users that were edited and filtered. For example, as Clara reported, although she was aware that all these photos that she makes a comparison to are photoshopped and edited, she cannot stop comparing hers to others’ weights. Having suffered from body image problems, Clara reported that her negative body image had not originated from using social media but was triggered and exacerbated by using social media. She expressed how distressed and insecure she feels about when she compares herself to others’ weights:

*“Really makes me feel awful like I said. I'd sit and cry to the point where my parents didn't like, my parents are so used to it that I would just break down out of the blue because of it.” (Clara, 15)*

#### Fear

Adolescents described their experiences of fear in various situations when using social media and their negative emotions about these experiences. For some participants, the sub-theme ‘fear’ captured experiences of fear of missing out on something/events or being left out by their friends:

*“I see a picture of like all my friends like going out and then like I haven't been invited. You know like that, that doesn't really make me feel, like, good” (Alice, 16)*

From the account of Clara, not being in contact with her friends for a day and delaying responding to her friends’ messages makes her stressed and cause a feeling of fear of being disliked and left out by her friends:

*“That can sometimes stresses me out because I think that they're gonna hate me for it or they're gonna dislike me because I've taken too long to reply to their messages.” (Clara, 15)*

Some adolescents reported they constantly check how many followers and likes they get on social media and check their social media accounts a few times before sleep to make sure they did not upload something to a story or post something by accident. From the account of Charlie, it is evident that not getting enough followers and likes on social media impact some adolescents’ mood and self-worth:

*“It would make you angry because you know, you're, you're following people that you know and they're not following you back. So it, what, what does that suggest, that they don't like you that, that much.” (Charlie, 16)*

#### Addiction/distraction

From adolescents’ accounts, social media have so many different contents that capture their interests, and the infinite scrolling feature of social media makes them addicted to it so that they feel they cannot live without it. The account from Charlie captures this theme:

*“We can't live without it. So, I think when, when we don't have it, it can create a longing urge for us to use it again.” (Charlie, 16)*

From some adolescents’ accounts, being addicted to social media and distracted by social media have a negative impact on their learning and education, sleep, and productivity during the day. These adolescents reported that social media is a tool they use for the purpose of passing time when there is nothing else to do and that they feel unhappy when they spend much time on social media and when they are not productive during the day.

## Discussion

The overarching aim of this study was to provide a rich interpretative account of the influence of adolescents' lived experiences on social media on their mental health during the Covid-19 pandemic. Participants’ experiences were organised into two main themes: the beauty of social media and the beast of social media. The analysis highlighted the range of positive and negative experiences that the adolescents had on social media as well as how they made sense of the impact of these experiences on their mental health during the Covid-19 pandemic. Positive experiences included connection and communication, exposure to positive content, positive social comparison, online social support, and resilience to stress during the Covid-19 pandemic whereas negative experiences consisted of exposure to negative content, negative social comparison, cyberbullying, fear and addiction/distraction.

The weight of evidence in each theme tended towards communication, entertainment, information, following, and monitoring others as the strongest motives for social media use. This was followed by ease of use, need to belong, content sharing, and connection, these identified motives were consistent with the previous study findings (e.g., Gray, 2018; Pertegal et al., 2019). It is likely that the positive effects of social media on adolescent mental health are linked to whether social media satisfies adolescents' needs, and motives for social media use. For example, as reported by adolescents in this study, connection and communication with friends make them feel better and happier, especially during the Covid-19 pandemic and national lockdowns where face-to-face communication was restricted. During adolescence, peer relations and approval are critical and social media seems to meet these needs. In addition, some adolescents in the current study expressed that scrolling through their feeds to see their friends’ posts, looking at funny images, watching funny videos, and seeing positive things happening in the world enhance their mood, which is consistent with the previous study findings (e.g., Fiacco, 2020; Radovic et al., 2017). This may be because such contents and activities satisfy adolescents' needs for entertainment and information. Online social support was another positive effect of social media that the current study findings revealed, and this is consistent with the previous findings (Fiacco, 2020; Ozbay et al., 2007; Zautra et al., 2010).

Although all adolescents engaged with both active and passive social media use behaviours, most adolescents preferred to remain passive on social media and monitor others. Active and passive use of social media may influence adolescent mental health in a different way. Previous study findings (e.g., Burnell et al., 2019; Thorisdottir et al., 2019) showed that passive social media use was positively associated with social comparison which in turn increased the level of anxiety and depression in adolescents. The present study revealed that adolescents who engaged with negative social comparison, especially female adolescents, were negatively influenced by social media. Making social comparison on high life standards make adolescents feel envy, and social comparison on weight and appearance makes them feel obsessed, anxious, and depressed. These findings are in accordance with findings reported by several studies (e.g., Nesi & Prinstein, 2015; Sun et al., 2016; Radovic et al., 2017; Wang et al., 2020; O’Reilly, 2020; Fiacco, 2020; Hjetland et al., 2021). However, contrary to this, we also found that social comparison has a positive impact on adolescent mental health. For example, in adolescents’ accounts, comparing themselves with someone who achieved something that they aspire to achieve inspires and motivates them towards their life goals. This is in accordance with the social comparison theory which indicates upward social comparison can motivate children and adolescents to improve themselves (Festinger, 1954). Our finding also supports a recent study finding of Pile et al. (2021) which identified that creating a future image helps adolescents to set intermediate goals and motivate them to achieve these goals. Similarly, making a horizontal social comparison, namely comparing themselves with those who look similar to them regarding physical appearance, and those who pass through similar experiences, makes them feel better, confident, and more secure. These findings suggest that social comparison influences adolescents’ mental health differently depending on the way they compare themselves and the way they perceive things; one may have negative feelings when they engage with social comparison while the other may not. One possible explanation for these mixed findings is the individual differences; perspectives and perceptions of two individuals looking at the same image may be different. Another explanation might be the fact that adolescents who are suffering from anxiety and depression are more likely to make social comparisons (Lyubomirsky & Ross, 1997), and social media may trigger adolescents' engagement with social comparison and make them feel even worse (Radovic et al., 2017).

The findings also revealed that negative content on social media that promotes animal cruelty, violence, discrimination, and negative news, make adolescents upset and frustrated. A similar conclusion was reached by other studies (e.g., Chao et al., 2020; Fiacco, 2020; O’Reilly, 2020). From the results, it was clear that being cyberbullied makes adolescents feel angry and sad. This is consistent with what has been found in previous studies (e.g., Nixon, 2014; Bottino et al., 2015; Radovic et al., 2017; O’Reilly et al., 2018; O'Reilly, 2020; Fiacco, 2020; Hjetland et al., 2021). The present study acknowledged the previous findings showing that both fear of missing out (e.g., Barry & Wong, 2020; O’Reilly, 2020; Hjetland et al., 2021) and addiction (e.g., Kelly et al., 2018; O’Reilly et al., 2018; Keles et al., 2020; Hjetland et al., 2021) negatively influence adolescent mental health. Finally, the results demonstrated that receiving few likes and followers causes adolescents to feel angry, sad and rejected, and have negative thoughts about themselves, which was also in line with the findings by Lee et al. (2020).

Turning now to the evidence on the implications of the Covid-19 pandemic, this study showed that the Covid-19 pandemic has increased the time spent on social media, and the frequency of online activities. These findings are directly in line with previous findings (e.g., Cauberghe et al., 2021; Fernandes et al., 2020), and can be explained by the fact that adolescents had nothing else to do when they stayed at home and were unable to go out and meet with friends face-to-face so that they spent more time on social media to satisfy their needs of communication, entertainment and information. From adolescents’ accounts, staying at home, having nothing else to do, being unable to go out and meet with friends due to the lockdown measures, exposure to news on the severity of the virus, the number of cases and deaths influenced their mental health negatively and made them feel worried, sad and bored. These reasons may explain the findings of previous studies (e.g., Cohen et al., 2021; Levita, 2020) that found an increase in depression and anxiety among adolescents who were healthy prior to the Covid-19 pandemic. Overall, in this study, it was highlighted that the Covid-19 pandemic and related contents/news had more negative impacts than positive; and that social media facilitated resilience to stress by satisfying adolescents’ needs for communication, entertainment, and information during the national lockdowns.

### Strengths and limitations of the study

This study has several strengths. First, the use of qualitative methodology with IPA study design enabled us to access rich, diverse, and in-depth information about the mental health implications of social media during the Covid-19 pandemic from the perspectives of adolescents, which has been underexplored previously. Second, as recommended by Smith et al. (2009), this study benefited from having a relatively small sample size, which allowed for an in-depth interviews and analysis to obtain rich personal data and where the voices of all participants were heard. Another strength lies in the heterogeneous sample composition in terms of age, gender, and the country of origin in order to reflect different perspectives and experiences. The age range of the adolescents was between 14 and 16, and the findings may be transferable to this age group. There was a gender balance among the eleven adolescents; five were female and six were male. Although the adolescents who participated in this study were selected across England, mostly from London, they were from different nationalities, and this diversity may have enriched the research results by providing different perspectives.

However, findings need to be read in the context of study limitations. First, the use of semi-structured interviews can be prone to interviewer bias. Although we attempted to minimise this by avoiding asking leading questions, participants may have responded my questions in a socially desirable fashion; they may have overrated or underrated their social media use and the implications of both social media and the Covid-19 pandemic on their mental health, which can then lead to biased responses and social desirability bias. Second, although every effort was made to enhance the rigor of analysis, qualitative analysis by its nature is subjective and as such researchers use their own interpretation of the data, which means that different perspectives and interpretations of the data are possible, and as such it is subject to interpretative bias.

### Implications for practice

Social media has both benefits and harms for adolescents. The findings of the present study, conducted during the Covid-19 pandemic, have implications for how authorities, parents and educators can support adolescents to enhance the potential benefits and minimise the possible harms of using social media.

The benefits of social media which were identified in the present study included exposure to positive content, positive social comparison, connection/communication, online social support, and resilience to stress. To maximize the benefits of social media, adolescents can be encouraged to follow/interact with social media accounts/pages that make them feel better, support them, have fun, motivate them and help them to learn. Engaging with social comparison is human nature and from the present study findings, it was evident that comparing oneself to others makes some adolescents feel motivated. As long as it is healthy and it makes adolescents feel happier/better, adolescents can be encouraged to use comparison as a motivating factor. Instead of feeling envious of others, they can be encouraged to focus on their own strengths, appreciate others’ achievements, and be motivated to achieve what others have achieved.

On the negative side, increased time spent on social media also increases the risk of cyberbullying, exposure to negative content, body image concerns, life dissatisfaction, fear of missing out, addiction, anxiety, and depression. It is the collective responsibility of parents, educators, and policymakers to protect adolescents from the harms of social media and guide them on healthy social media usage. There are several actions that can be taken to prevent adolescents from the potential harm of social media. Firstly, by educating not only adolescents but also parents/caregivers, and educators about the harms of social media, the harmful effects of social media can be mitigated. It would be beneficial if schools have media literacy education in their academic curriculum. Media literacy education can help adolescents to be aware of how social media algorithms and bots work, misinformation, fake news, and unrealistic images, what they may experience on social media, and how these experiences can impact their mental health. Parents and educators should communicate with adolescents in a friendly, open, and non-judgmental manner to help adolescents to open up about what they experience on social media and how they feel about it. Some adolescents may have mostly positive experiences on social media while others may have mostly negative. Therefore, parents and educators should not be too ready to blame social media and should also be able to acknowledge the benefits of social media.

Adolescents should be encouraged to talk to their parents and/or teachers about any experiences of cyberbullying and adolescents should be educated about what is right and what is wrong and discouraged to bully others online as well. Some parental control apps can help parents to monitor what their children are doing online, their social media activities (e.g., posts, likes, comments, messages, etc.), and browsing history. By monitoring their children’s social media accounts, parents can identify any potential danger and cyberbullying acts toward their children or against others from their children. In addition, using social media in a public setting may increase the risk of being a victim of cyberbullying. Therefore, adolescents should be encouraged to make their social media accounts private and contact people they know in person only.

Adolescents can experience body image concerns, negative self-image, eating problems, and life dissatisfaction as a result of social comparison on social media. Adolescents should be made aware that social media is full of unrealistic, edited, and photoshopped images. Also, school-based detection and interventions should be developed to identify adolescents at risk of mental health problems and improve adolescents’ self-image, self-esteem, and life satisfaction. Adolescents should be encouraged to unfollow/block social media accounts that may trigger social comparison and make them feel unhappy and worthless.

Adolescents may experience fear of missing out when they are not on social media and thus they may spend much time on social media, which may also impact their sleep patterns. Parents should set limits on their children's daily time spent on social media, and discourage late-night electronic device use by asking them to keep their phones/tablets away from their bedrooms. Adolescents should also be encouraged to engage with offline activities such as sports and face-to-face contact with family and friends. As actions speak louder than words, parents should not only set rules for their children but also be a role model by being not occupied with their phones during family gatherings. By limiting daily screen time, the risk of being addicted to social media may be mitigated as well.

Finally, teachers should closely observe adolescents regularly to detect any changes in their behaviours. When they observe any suspicious behaviour other than normal, they should contact the parents/caregivers and the school-based counsellor. Likewise, parents should talk to their children about how they are feeling and listen to them in a non-judgemental manner. If they suspect that their children have mental health problems, parents should seek professional help for their children. In this way, any mental health problems can be detected and treated at an early stage.

### Implications for future research

This study has highlighted several potential directions for future research. The findings clearly illustrate that adolescents’ experiences of social media and the impact of these experiences on their mental health are multifaceted. Although some experiences have solely negative impacts or solely positive impacts, other experiences have both negative and positive impacts. For example, it was observed that the impact of social comparison on adolescent mental health was either positive, negative, or no effect depending on the way they compare themselves to others and individual differences. Future research aimed at exploring individual differences in the relationship between social comparison on social media and mental health in adolescents might be beneficial. Results also suggest that the Covid-19 pandemic had more negative effects than positive on adolescent mental health and that social media helped with coping with the negative impacts of the Covid-19 pandemic and restrictions. Adolescents emphasized the importance of connection and communication, entertainment, learning what is happening in the world, following and monitoring other people’s life and they perceived social media as a helpful and powerful tool that allowed them to access these during the Covid-19 pandemic when restrictions were in place. This illustrates that social media may have acted as a protective factor against the negative impacts of the Covid-19 pandemic and restrictions, which may be worth investigating in future research. Findings also indicate that the Covid-19 pandemic has increased their time spent on social media and that adolescents preferred to remain passive on social media rather active use. This illustrates that increased time spent during the Covid-19 pandemic increase the passive social media use activities among adolescents. Passive social media use is often linked to monitoring others’ activities which may then increase the likelihood of exposing negative contents, engaging negative social comparisons, and experiencing FOMO. It would also be beneficial if future research explores the mediating/moderating effect of protective as well as risk factors identified in this study (e.g., negative/positive social comparison, fear, exposure to positive/negative contents, cyberbullying) on the relationship between social media use and mental health outcomes in adolescents. Finally, perspectives and experiences of adolescents with mental disorders may differ from the sample in this study. Future research should also consider exploring the experiences of adolescents with a diagnosed mental disorder to compare and contrast the results reported in this study.

## Conclusions

This study showed that the adolescents had a range of negative and positive experiences with social media and mostly negative experiences with the Covid-19 pandemic. However, social media has had mostly positive impacts for many adolescents during the Covid-19 pandemic and the national lockdown by satisfying adolescents’ needs for communication, entertainment, and information. The results also suggest that the mental health implications of social media are multifaceted and are likely to differ from one individual to another. This study may inform future research and deepen and enrich their knowledge in the field about the adolescent's perceptions and experiences of social media and the Covid-19 pandemic. Further investigations may be needed to better understand the individual differences in the relationship between social media use and mental health.

## Data Availability

A confidentiality agreement with participants prevents us from sharing the dataset.
